# A Miscarriage Between the Fourth and Fifth Months

**Published:** 1893-04

**Authors:** Samuel A. Kimball

**Affiliations:** Boston, Mass.


					﻿PUERPERAL CONVULSIONS.
R. E. Jameson, M. D., Jamaica Plain, Boston, Mass.
Mrs. A., aged thirty-two, of a decidedly psoric constitution.
Mother at climacteric season for four years and died of insanity.
Her aunt, sister to her mother, died of puerperal convulsions
at thirty-two. When eighteen was thrown from a carriage and
spine injured. Suffered for several years with uterine disease,
and received all kinds of local treatment at the hands of so-
called homoeopathic physicians. In January, 1882, gave birth
to a daughter, and was in poor health for several years. Be-
tween the birth of her daughter and 1890, when I first became
acquainted with her, had two miscarriages. In 1890 became
pregnant again and called me in to relieve her of various symp-
toms incident to her condition, and- prevent if possible another
miscarriage. She had a good deal of leucorrhoea, constipation,
headaches, and indigestion, with vomiting. Her headache and
the gastric symptoms were aggravated by motion, and I gave her
Bryonia®”1, which relieved all the symptoms but the leucorrhoea.
For that I gave her Puls, and Sepia, which helped it very
much but did not entirely cure it. She went to term and was
delivered of a girl, that lived only twenty-four hours. She
became pregnant again, as she was very anxious to have chil-
dren, and had a much more comfortable time than at any pre-
vious pregnancy. April 4th last I was called about half-past
twelve a. m. As she had previously had tedious labors her
husband, who summoned me, did not hurry very much, and
when I arrived I was very much surprised on being told that the
baby had arrived. She had had a very easy labor, and was
feeling very happy over the advent of the boy. I visited her
again at half-past nine and found her comfortable with the ex-
ception of headache, which she said was not very bad and which
did not alarm me, as she was so subject to headache. I left a
remedy for it, however. At eleven o’clock I called at my house
and was told by my wife that I was wanted at once at Mrs. A.’s,
that she was in convulsions. I drove at once to her and found
her in a violent spasm, the fourth that she had had. Her face
was of a bluish color, there were twitchings of the muscles, es-
pecially of the face and eyelids; with great restlessness, tossing
from one side of the bed to the other, with constant delirium.
I gave her Hyoscyamus. In two hours there was apparently a
little improvement in that she became conscious, and said she
felt very uncomfortable and wanted to pass water. The improve-
ment did not last, however, and I sent for Dr. Wesselhoeft.
The symptoms changed, there were now stupor, stertorous
breathing, rigidity of limbs, head turned to right, eyes turned
up, pupils dilated, and nearly insensible to light; pulse rapid ;
throws arms out at right angles with body, frothing at mouth,
face livid and bloated, tongue swollen and abdomen very
much distended and tympanitic. Hot sweat. I gave Opium,
which was approved by Dr. Wesselhoeft. Dr. Kimball came
out at night, and remained till morning. We went over the
symptoms together very carefully, and could find nothing better
indicated than the Opium. In the night, however, the symp-
toms changed again. The stertor gave place to a constant moan-
ing between the spasms. The spasm began in the right eyelid,
and the right side was convulsed, while the left was motionless,
as though paralyzed. The hot sweat continued, and there was
rolling of the head on the pillow. Dr. Kimball gave Bell.om. The
spasms were not quite so frequent, and from 5.30 to 8.30 she
had none; between that and 11.30 she had five. I repeated the
Bell, at 8.30 and at 10.30. At 11.30 she had the last one.
Then respiration ceased and the pulse could scarcely be felt,
and I thought she was gone. She remained unconscious till
Wednesday night, about thirty hours after the convulsion ceased,
when a violent delirium set in, and it was difficult to keep her
in bed. Her screams could be heard over the neighborhood.
I gave Coffea, Hyoscyamus, and Stramonium, as they seemed
indicated. On Saturday her husband said her mental condition
seemed like anger, real ugliness. I did not wonder that he
thought so, for she struck him several times in the face. I asked
him if she had shown any such disposition during her preg-
nancy or during labor. I had never seen anything of it, and
had not been told of any such symptoms. He answered, “ Oh I
yes.” The little daughter came to him one day and wanted
him to take her somewhere because mamma treated her so, and
sometimes it seemed impossible to do anything to please her.
Saturday night or Sunday morning I gave her Chamomilla.
Sunday P. M. I called, and found her in a quiet sleep. In the
evening I called again, and she was still sleeping, though she
had awakened once or twice and taken nourishment. From that
time she improved, and is now perfectly well.
Mastitis.
Mrs. F., about two weeks after confinement, called me in to
see her breast, which was somewhat inflamed, but not very
painful. It was on the right side, and the inflammation was of
a pale red. I gave Bryonia, and in a few days called again and
found it worse; the inflammation had increased and extended
in area. She said the pains were pricking in character. It looked
very much as though an abscess was inevitable. But she had
another symptom. She said she was lame when she sat down,
and it hurt her to rise from her seat. She was also constipated,
had no stool without an enema. I gave her Lycopodium'”1, two
doses. The abscess was averted, the constipation relieved, and
the ischiatic lameness disappeared with the rest.
Discussion.
Dr. Plummer—I had a case of convulsions strikingly similar
to the one Dr. Jameson has reported, and occurred at very
nearly the same time. In my case as in his, Hyoscyamus
seemed indicated, yet did not help; but Opium gave marked
relief.
It was, however, insufficient, and had to be followed by
Belladonna.
My patient had paralysis of the right side following the at-
tack, which cleared up in the course of ten days or two weeks
under Phosphorus, and at the end of four weeks she was entirely
cured.
Dr. Carr—Does the doctor recall the character of the pulse at
the time of the first headache, whether it had the globular feel-
ing described by Dr. Gregg? The headache which preceded the
convulsions, I mean. It has been my experience that when on
the second or third day such a pulse and headache occurs, it is
almost a characteristic indication for Belladonna. I think that
probably Bell, would have averted the first convulsion if given
as soon as indicated by that pulse.
Dr. Kimball—During the night I frequently took the pulse,
and often could not find it at all. She would have a convul-
sion ; then in an hour another one; then one in fifteen minutes;
then in forty-five minutes. There would be, in other words, a
long interval and then a short one. After the Opium she
moaned, instead of being in a stertorous sleep. After the dose
of Bell, the intervals began to increase, and by morning she was
having two hours between the convulsions.
Dr. Long—There is one part of that paper which impresses
me very deeply, and makes me feel sad to know that in such
extreme cases I stand entirely alone. I have no Dr. Wessel-
hoeft or Kimball to help me out. There are many Hahne-
mannians who have to meet the severe strain of their terrible
cases alone, unhelped by the friendly hands of their professional
brethren. Some of you who are more fortunate do not realize
how awful it may sometimes be to stand before a terrible case of
sickness, unhelped save by your God and your materia medica.
FIBROID TUMOR OF THE UTERUS.
E. T. Adams, M. D., Toronto, Can.
Mr. President and Members :—I have briefly to mention
some peculiarities occurring in the case of a patient suffering
from fibroid tumor of the uterus, and which I reported in
extenso at our last meeting.
Perhaps some of you will remember the very satisfactory
results obtained by the use of Lil-tig.em in checking the pro-
fuse floodings. Monthly discharge became as natural as for many
years—every three weeks and lasting four days. The tumor
ceased to grow—then began to diminish and continued to di-
minish. The woman became natural and normal in her mental
condition. In fact, patient like, a common remark with her
was : “ I doubt whether I ever had a tumorand as improve-
ment went on “ I don’t believe I ever had a tumor.” Such was
the happy state of affairs till the middle of November last, when
one midnight a messenger delivered a telegram stating that an
only and much loved sister had died very suddenly. The
shock from the sudden news and grief at its cause affected her
very severely. In three days you would scarcely have recog-
nized the woman. She returned from the funeral and in about
two week called on me.
She was in a most despondent state, similar to that of the
year before. Thought she was going mad—was confident peo-
ple would notice that there was something wrong with her
mentally. Was sure some fearful misfortune was about to
happen. Was confused, forgetful, no life or energy, no inclina-
tion for business—love of which, when well, was as the breath
in her nostrils—anxious and given to spells of weeping. Was
much bloated, and felt sure that the tumor was again growing.
I gave her Lil-tig.®"1 again, and at the end of two weeks found
but little improvement—possibly a slight relief mentally. At
this visit she was sure the tumor was again growing, and she
was confident the growth now was on the opposite side of womb.
On examination this proved correct. The tumor was rapidly
developing, while on the side originally affected, I could dis-
cover no enlargement. At this time she also complained of
numbers of small warts over her upper abdomen, and on upper
left chest and shoulder. Lil-tig. had evidently done all it could,
and after careful comparison, I gave Calc-carb.20, three doses,
two hours apart, and Placebo.
In a couple of weeks she returned and mentally was much
better, brighter, and more cheerful, with desire to attend to her
business returning. Quite a satisfactory change, and she re-
ceived a supply of Placebo.
The improvement continued gradually and all round. Two
weeks later she felt sure that growth in tumor had ceased, and
she began again to expect to be a well woman. Some time in
February improvement seemed to cease, after a severe cold. By
the way, disposition to take cold easily was a prominent con-
dition in the beginning of this her second attack, and I gave her
Calc-carb.om, one dose. This was the last active medicine she
received. She has been comparatively well all the past spring,,
and as she begins to “ wonder whether she had a tumor at all,”
I regard her prospects as favorable though uncertain, but that
this may prove a perennial root and thus afford me an annual
branch on which to address you.
To me this case has been very peculiar. No doubt could
exist as to the presence of a tumor in 1890 and early part of
1891. It was so diagnosed by several gynecologists—the pro-
fuse hemorrhages, etc.
And no doubt can exist that she was on the road to health if
not cured prior to the shock, grief, etc. Then the sudden return,
and on the opposite side of womb. I cannot account for this.
It’s peculiar.
But one thing prevents my hoping too much from past suc-
cess in this case, and that is that rheumatism (muscular), from
which she has suffered for years, has not been to any appreciable
extent even relieved by the treatment.
On the other hand, she keeps a medicine chest with remedies
in low potencies with which she doses herself for cold and
slight attacks, only sending for the doctor when either she or
her friends get alarmed. But the drugs she took did not in
any way seem to interfere with the action of the CM potency.
Possibly they did with the 2c.
I neglected to say that the crop of warts is disappearing
rapidly.
Discussion.
Dr. Johnson—One point Dr. Adams brought out which I
think is important, namely, that remedies in lower potencies do
not afiect the action of a higher potency. I have noticed it in
several cases. When a patient who is under the action of a
constitutional remedy has an acute attack we may give an ap-
propriate remedy in a lower potency without affecting or dis-
turbing the action of the higher.
Dr. W. L. Morgan—I have observed the same thing, that
the lower potencies do not interfere with the higher. This case
reminds me of one I had five years ago, a fibroid tumor. The
patient had had a large one taken away nine years before, and
this was still larger than the one taken away. It was so large
that when it came down in the pelvis I could not get my finger
in the vagina above it. It interfered with the flow of urine.
It was exceedingly hard and very tender to touch. Her symp-
toms called for Sepia. I gave the 200th. In three months it
removed the entire tumor, and it remained absent for a long
time. Returning again in six months Sepialm removed it again.
An old laceration of the cervix only remained, until last fall
she went into a hospital and had an operation. After that came
prolapse without return of tumor, for which she came to my
office a few days ago. She had been wearing a pessary. I gave
her Sepiacm, and have not seen her since. That was just before
leaving home for this place. She was much improved two
months later.
HYDRO-SALPINGITIS. A CASE CURED WITH
GELSEMINUM.
J. H. Allen, M. D., Logansport, Ind.
Case.—Mrs. E., aged twenty-seven, light blonde, above
medium height, and weight about one hundred and forty, came
to me one year ago for an examination and treatment for a large
growth in the left abdominal region. Pregnancy had been
diagnosed, with advice to rest as much as possible, especially
during the menstrual period, which function had kept up with
clock-like regularity every month. During the inter-menstrual
period she suffered very little pain with exception of an occa-
sional dragging or bearing-down feeling in the region of the
uterus. As the usual period of gestation had passed she became
very anxious to know what the trouble was. After examining
her case carefully I made a diagnosis of some tubular trouble,
and that of cystic nature and in all probability hydro-salpin-
gitis.
Though it is hard to diagnose these cases, yet by taking into
consideration that the case had a gonorrhoeal history, together
with the location of tumor, the consistency of the walls of the
growth and by the aid of percussion and the bimanual ex-
amination, together with the large amount of serum that passed
at the close of each menstrual period I made it pretty conculsive.
The tumor was then the size of a child’s head or larger, mobile
and fluctuating, the os was patulous, the genital organs showed
marked signs of pregnancy and in an advanced stage, morning
sickness had continued all through her illness, the breasts had
increased in size and were fully as large as in the eighth month.
She was now suffering considerable pain in the region of the left
ovary, which was probably due to pressure or distention caused
by the accumulated fluid, as it was worse just before the
menses and better after the profuse flow of serum that came on
at the close of the menstrual period.
I called in an eminent surgeon from a distant city in consul-
tation, who advised an operation for removal of the ovary as
well as the diseased tube. I did not agree with him as to his
mode of procedure, and my patient would not listen to it for a
moment. I therefore concluded to try my remedies and await
results. On examination of the symptomatology of her case the
following symptoms were noted: A feeling of fullness and
heaviness in the uterine region, spasmodic, cramp-like pains
during the menses, sharp pains running from uterus to back
and hips. A languid aching in back and hips a day or so
before the menses, great weakness and loss of power in the
lower extremities, very little pain after the menses begin ; she
says that her pains resemble false labor pains, when she gets
tired she has a lump in the throat which she cannot swallow.
After the menses suffers with pains in back of head and spine,
pains running up the back of the neck, with a feeling of tight-
ness in the brain, she is irritable and easily angered. There is
considerable fever in the afternoons and evenings accompanied
with twitching of the muscles. The menses last eight days,
for the first three days they seem quite natural, but during the
rest of the flow it is very light-colored and resembles serum.
Gelseminum was given in the one thousandth potency, which
relieved her menstrual pains, and as she continued to improve
and feel better generally I did not change the prescription. In
ten days after receiving the remedy the flow of serum increased
to almost a pint per day for a week, when it gradually decreased
and finally ceased entirely. The next menstrual period only
lasted five days, she experiencing very little pain and no return
of the flow of serum. In all six powders of the remedy were
given during the period of two months, usually a powder was
prepared in water and given every hour until she found relief
when it was discontinued. Since then she has received no
medicine, though it is nine months since I discharged the case.
At the end of the third month all symptoms of the tumor had
disappeared, and the menses resumed the normal again, and she
has been in perfect health since.
HEMORRHAGE FOLLOWING ABORTION READILY
CONTROLLED BY THE INDICATED REMEDY.
J. A. Tomhagen, M. D., Philadelphia, Pa.
Case I.—Mrs. E. H., set. twenty-four. Menses absent two
months and positive she was pregnant. Has taken many drugs
and teas to rid herself of the foetus. March 4th, 1889, she rode
a horse about three miles, and noticed that she was flooding
before she reached her destination. She was put to bed, and after
several severe pains the foetus was extruded. The hemorrhage
was profuse and painless and bright red. When I arrived her
face was blanched, and she frequently gasped for breath. The
slightest motion would cause a gush of blood, which was fol-
lowed by a deathly sinking at the epigastrium.
Ipec.200 controlled the hemorrhage at once. The woman ex-
perienced a peculiar, indescribable sensation pass over her in a
few seconds. The gushes then came at longer intervals and
the fainting ceased immediately. The one dose was all-suffi-
cient.
Case II.—Mrs.' F. G., set. twenty-eight. Blonde. This
woman maintained that the “ miscarriage ” came on spon-
taneously ; she could account for it in no way except that carrying
a bucket of water caused it. She had been flooding in spells
for several days, when I saw her, May 13th, 1889. Blood dark
and clotted. As soon as she saw me, she began to weep, but
could say nothing; moreover, she was as patient as a lamb; her
mouth was parched, and yet she desired no water. The foetus
of a few weeks must have been buried with the clots since I
could find none. Puls.2m.
In five minutes a sensation as if the whole external
genitals were drawn up into the pelvis followed. (Puls.10m has
produced this several times. In fact, one girl remarked that I
had given her dynamite, the action was so violent.) With this
sensation the hemorrhage ceased and returned only slightly in
six hours. No more medicine.
Case III.—Mrs. J. S., set. twenty. Married a short time;
got into an altercation with another of the fair sex. Both being
typical brunettes, they well-nigh demolished each other. My
patient tore and swore, fumed and stamped, when separated,
because she was not permitted to annihilate her adversary; she
retired very disconcerted, and during the night was taken with
violent cramps and vomiting of bile; intense bearing-down
pains and sharp, excruciating pain in the left ovarian region ;
she could not lie still a moment, and the only relief was obtained
from hard pressure and hot fomentation on abdomen. The
hemorrhage was slight, two A. M., Coloc.3m.
In twenty minutes the pains abated and hemorrhage stopped.
Six a. M., very comfortable, but face as yellow as a lemon ;
no more pain and carried child to full term.
Remarks.—The single dose of the appropriate remedy was all-
sufficient in these cases.
Some maintain that it is criminal to rely on medicine in
abortion. It is criminal to do other than give the indicated
remedy.
The above cases I regard as typical of abortion.
Am., Gels., Sabina, Apis, etc., do the work as effectually,
when called for.
China often removes membranes that have kept up flooding
for weeks, and then the hemorrhage ceases.
Pyrogen, cured several cases where symptoms of septicaemia
supervened. The China and Pyrogen, cases come from the
scientific doctors.
Dynamics supersede mechanics.
ALLOPATHIC SUPPRESSION OF LOCHIA.
D. C. McLaren, M. D., Ottawa, Can.
This case is that of a lady about thirty-five years of age,
whose second child was born on August 1st, 1885. Up to this
date she had always been a strong, healthy woman, but, after a
'few days, the foul condition of the lochia excited the combined
animosity of physician and nurse, and an intra-uterine injection
of Iodine was ordered and faithfully administered. This
promptly dried up the discharge, but was immediately followed
by a sense of fullness in the abdomen ; this sensation soon became
reality, and for some months she was as much distended as if
again pregnant. Severe headaches came on in November, for
which she was treated by the allopathic methods in vain. Finally,
a specialist discovered a lacerated cervix, and operated. The
headaches left, but in place of them came a most distressing op-
pression across the chest; this was so bad at one time that she
was almost out of her mind. This trouble was at its height in
March, 1890, when she had a miscarriage. She came to me in
October, 1890, weeping bitterly as she described her symptoms,
saying she surely had heart disease, that she was possessed by
the devil, etc. She complained so much about her heart that it
was examined, but found to be sound ; there was, however, a
decided throbbing at the pit of the stomach, which was at times
visible externally. This spot was exceedingly tender on pres-
sure, and there seemed to be a lump beneath. I hesitate, how-
ever, to declarethat it was an aneurism, because, in our provings
of Iodine, there occurs the symptom : “ Violent throbbing of
the abdominal aorta.” I tried several apparently indicated
remedies, but Lycopodium was the first to do any real good ; its
principal indication was the formation of flatulence in the
stomach and relief from eructation. The course of the treat-
ment was interrupted in March, 1891, by another miscarriage, for
which the lady’s husband, in his haste, called in the former allo-
pathic physician. During the summer I gave one dose of
Iodum high, but cannot record any great improvement from it.
I became convinced that pregnancy would do this case much
good, and when she was again threatened with miscarriage, in
October, I was promptly on hand, and stopped the manifestation
with a single dose of Sabina200. Subsequent symptoms required,
from time to time, a dose of Belladonna, but during the last three
months of pregnancy she was left under the influence of Psori-
num. Her baby was born on Good Friday, 1892, and the lochial
discharge for a week was most astonishingly profuse. She recov-
ered well, and the pulsation at the stomach pit has entirely
disappeared. Ido not claim to have cured a case of aneurism, but
to have in some degree overcome the results of the original allo-
pathic suppression.
Discussion.
Dr. Farley—The trouble in that case suggests to me a case '
I had last week. A physician in my town asked me to see a
case for him, which he called asthma. He had given several
remedies. She had long been a sufferer from hemorrhoids, and,.
I judge, also a fissure. She had been operated on for rectal
trouble, and soon after had the asthma. I gave Kali-bichrom.200,
in water, at intervals of two hours for twenty-four hours. The
respiration was better, and she was sittingerecton the next day.
It was a marked improvement, but her rectum was getting sore,,
and in a short time her asthma was gone, but her rectum was
again the seat of much suffering. The character of the expector-
ation drew my attention to the remedy.
The following amendment to the By-Laws was presented by
Dr. Bell to be acted upon at the next meeting :
“ The order of business may be changed at any annual meet-
ing by a unanimous vote.”
The following amendment to the By-Laws was presented by
Dr. Custis to be acted upon at the next meeting :
“ The Association shall meet annually at such time and place
as may be fixed upon by ballot at the annual meeting. If suit-
able arrangements cannot be made in accordance with the in-
structions given, the Executive Committee may, with the ap-
proval of a majority of the members, change the place of meet-
ing. The ballot to be taken by mail, the members to have the
choice of two places named by the committee.”
The motion was made and carried that the Executive Com-
mittee have charge of the publication of the Transactions.
A vote of thanks was passed to the proprietor of the hotel.
The minutes of the meeting were read by the Secretary and
were approved.
Adjourned sine die at 11.30 a. m.
				

